# Adipose tissue metabolic and inflammatory responses to a mixed meal in lean, overweight and obese men

**DOI:** 10.1007/s00394-015-1087-7

**Published:** 2015-10-29

**Authors:** Rebecca L. Travers, Alexandre C. Motta, James A. Betts, Dylan Thompson

**Affiliations:** 1 Department for Health, University of Bath, Bath, BA2 7AY UK; 2 Unilever Food & Health Research Institute, Vlaardingen, The Netherlands

**Keywords:** Adipose tissue, Metabolism, Inflammation, Postprandial, Human

## Abstract

**Purpose:**

Most of what we know about adipose tissue is restricted to observations derived after an overnight fast. However, humans spend the majority of waking hours in a postprandial (fed) state, and it is unclear whether increasing adiposity impacts adipose tissue responses to feeding. The aim of this research was to investigate postprandial responses in adipose tissue across varying degrees of adiposity.

**Methods:**

Thirty males aged 35–55 years with waist circumference 81–118 cm were divided equally into groups categorized as either lean, overweight or obese. Participants consumed a meal and insulinaemic, glycaemic and lipidaemic responses were monitored over 6 h. Subcutaneous adipose tissue samples were obtained at baseline and after 6 h to examine changes in gene expression and adipose tissue secretion of various adipokines.

**Results:**

Following consumption of the meal, insulin and glucose responses were higher with increased adiposity (total AUC effects of group; *p* = 0.058 and *p* = 0.027, respectively). At 6 h, significant time effects reflected increases in IL-6 (*F* = 14.7*, p* = 0.001) and MCP-1 (*F* = 10.7, *p* = 0.003) and reduction in IRS2 adipose tissue gene expression (*F* = 24.6, *p* < 0.001), all independent of adiposity. *Ex* *vivo* adipokine secretion from adipose tissue explants remained largely unchanged after feeding.

**Conclusions:**

Increased systemic measures of postprandial metabolism with greater adiposity do not translate into increased inflammatory responses within adipose tissue. Instead, postprandial adipose tissue changes may represent a normal response to feeding or a (relatively) normalized response with increased adiposity due to either similar net exposure (i.e. per g of adipose) or reduced adipose tissue responsiveness.

**Electronic supplementary material:**

The online version of this article (doi:10.1007/s00394-015-1087-7) contains supplementary material, which is available to authorized users.

## Introduction

Adipose tissue expansion is accompanied by a more pro-inflammatory gene expression profile which, together with increased secretion of inflammatory cytokines, may contribute to systemic low-grade inflammation and insulin resistance [1]. The vast majority of what we know about adipose tissue is restricted to observations derived after an overnight fast. However, adipose tissue is a dynamic player in overall metabolic regulation, and it cannot be assumed that fasted measurements will adequately reflect the overall role and function of adipose tissue, particularly since the majority of the waking day is spent in a fed (i.e. postprandial) state.

Obesity typically increases postprandial glucose, insulin and triglyceride responses in blood [2, 3], and it is reasonable to anticipate that this could alter the adipose tissue responses to a meal. Surprisingly few studies to date have investigated the presence of postprandial inflammation within adipose tissue in humans, typically comparing postprandial responses to differing qualities/quantities of lipids in people with metabolic syndrome [4] or comparing responses in (non-obese) relatives of people with type 2 diabetes versus controls [5]. In both these studies, significant increases in adipose tissue gene expression of a number of inflammatory genes including MCP-1, TNF-α, IL-1β and IL-6 were detected, suggesting that there may indeed be postprandial inflammatory responses in adipose tissue [4, 5]. Remarkably, there has been no attempt to compare postprandial responses within adipose tissue between lean and obese individuals. Thus, it is unclear whether these changes in inflammatory markers in adipose tissue represent a ‘normal’ phenomenon or something that is influenced by increasing adiposity, as is the case with other more systemic changes (e.g. insulinaemia). This is central to our understanding of obesity-related inflammation and chronic disease.

The aim of this research was to investigate whether the greater postprandial metabolic response (i.e. hyperinsulinaemia) observed with increased adiposity leads to an increased inflammatory response within adipose tissue in humans.

## Methods

### Experimental design

Thirty men aged between 35 and 55 years were recruited by local advertisement and following preliminary anthropometric assessment and were classified according to waist circumference as lean ≤94 cm, overweight 94–102 cm and obese ≥102 cm [6]. Recruitment was on a first-come first-served basis and took place between September 2011 and April 2012 until there were 10 participants in each waist circumference category. Participants attended one main trial in the Physiology Laboratories at the University of Bath, in which blood and adipose tissue samples were obtained before and 6 h after consumption of a mixed meal with further blood samples taken at regular intervals throughout. Primary outcome measures included examination of changes in adipose tissue gene expression and secretion of selected cytokines from adipose explants after the meal according to levels of adiposity (and postprandial metabolic responses in blood). This trial forms part of a larger investigation in which immune cells present in adipose tissue were characterized and their relationships with baseline adipose tissue inflammation examined, as has been previously reported [7]. All procedures followed were in accordance with the protocol reviewed and approved by the National Health Service South West (Southmead) Research Ethics Committee (11/SW/0193) and have therefore been performed in accordance with the ethical standards laid down in the 1964 Declaration of Helsinki and its later amendments. This trial is registered at ClinicalTrials.gov (ID: NCT02416843), and all participants gave their written informed consent prior to their inclusion in the study. Individuals were excluded from participation if they had a history of diabetes, cardiovascular disease or dyslipidaemia, were taking any medications known to interfere with immune function or lipid/carbohydrate metabolism, if they smoked or had not been weight stable for >3 months (i.e. weight change >3 %; [8]). In addition, participants were excluded if they reported food intolerances/allergies to any component of the meal (e.g. dairy or wheat) and habitually performed more than 6 h of vigorous-intensity physical activity or 10 h of moderate-intensity physical activity per week, assessed via a self-report questionnaire (as these individuals would be unlikely to fit the meal standardization procedure). Participant characteristics within the categories lean, overweight and obese based on measurements of waist circumference are given in Table 1.

**Table 1:** Descriptive statistics of participants classified according to waist circumference

Classification based on waist circumference	Lean *n* = 10	Overweight *n* = 10	Obese *n* = 10	One-way ANOVA *p*
Age (years)	44 (2)	48 (2)	45 (2)	0.218
Height (m)	1.78 (0.02)	1.77 (0.02)	1.81 (0.03)	0.469
Body mass (kg)	74.8 (1.6)	83.7 (1.8)	100.2 (3.3)	<0.001
Body mass index (kg/m^2^)	23.6 (0.6)	26.7 (0.4)	30.7 (0.9)	<0.001
Waist circumference (cm)	87.0 (1.4)	97.7 (0.8)	109.4 (1.8)	<0.001
Fat mass index (kg/m^2^)	4.5 (0.3)	6.9 (0.2)	9.5 (0.6)	<0.001
L1-L4 fat (%)	19 (1)	30 (1)	37 (2)	<0.001
Resting metabolic rate (kcal/day)	1644 (65)	1722 (55)	1882 (74)	0.045
PAL^I^	1.94 (0.10)	1.66 (0.06)	1.67 (0.10)	0.066
Energy consumed in test meal (kcal)	1063 (42)	1113 (36)	1217 (48)	0.045
Fasting glucose (mmol/L)	4.4 (0.3)	4.8 (0.3)	5.3 (0.2)	0.043
Fasting insulin (pmol/L)	27.3 (4.8)	39.6 (7.0)	59.5 (10.1)	0.020
Fasting triglycerides (mmol/L)	0.9 (0.1)	1.3 (0.2)	1.0 (0.1)	0.123
Fasting NEFA (mmol/L)	0.33 (0.04)	0.48 (0.13)	0.43 (0.05)	0.469

Mean (SEM) values shown and statistical differences between the three groups were assessed by one-way ANOVA, *p* values shown. Abbreviations used: L1-L4 = central fat estimated between Lumbar regions 1–4 using DEXA; PAL = physical activity level which is the product of total energy expenditure ÷ basal metabolic rate (^I^lean *n* = 9); *NEFA* non-esterified fatty acids

#### Sample size determination

The sample size for this study was determined using a clinically relevant difference in serum insulin response to a meal between lean [AUC 93 µU/mL (±29 SD)] and obese [AUC 169 µU/mL (±51 SD)] individuals with an effect size of 1.8 (G*Power 3.1.5, Germany) [9]. Thus, with 95 % power and 5 % alpha, 18 participants (nine lean and nine obese) would be required to detect a statistically significant difference in serum insulin AUC between groups.

### Preliminary measurements

Participant waist circumference, body mass and height were measured and body mass index calculated [BMI; mass (kg)/height^2^ (m^2^)]. Participants were fitted with a combined heart rate and accelerometry monitor (Actiheart) for a period of 9 consecutive days to determine habitual physical activity level (PAL; total energy expenditure/basal metabolic rate [10]). Participants were asked to maintain their normal lifestyle habits/routines during this period with the first 2 days of activity monitoring being excluded from analysis to account for potential reactivity [11].

### Main trial

Participants were asked to refrain from performing any strenuous physical activity for 48 h and consuming alcohol/caffeine for 24 h prior to testing. Trial days were scheduled so participants had been free from any self-reported illness for a minimum of 2 weeks in order to reduce immune system disturbance. Participants arrived in the laboratory in the morning after an overnight fast (minimum 10 h) and after consuming 1 pint of water upon waking. Body mass (post-void) was determined using a digital balance (Tanita Corp., Japan) with participants wearing lightweight shorts. Dual-energy X-ray absorptiometry (DEXA; Discovery, Hologic, Bedford, UK) was used to estimate lean and fat mass. Central adipose tissue (abdominal subcutaneous and visceral adipose tissue) was estimated from a central region between L1 and L4 which has previously been shown to be comparable to estimates of central adipose tissue measured by computerized tomography (CT) [12] and correlates with measures of metabolic health [13]. Fat mass index (FMI) was calculated using the equation FMI = total fat mass (kg)/height^2^ (m^2^) and interpreted using ranges that match the WHO BMI classifications [14]. Indirect calorimetry was used to estimate resting metabolic rate (RMR) after participants had rested supine in bed for 10 min [15]. This value was used to adjust estimates of total energy expenditure and PAL and to calculate the energy requirements for the test meal given to participants.

#### Meal composition and energy requirements

Participants were given a breakfast meal relative to their resting metabolic rate comprising brioche, strawberry jam (both Sainsbury’s, UK), margarine (Stork, Unilever), milkshake (fresh chocolate milk and whipping cream; both Sainsbury’s, UK, with added icing sugar; Silver Spoon) and a cup of decaffeinated tea (PG tips, Unilever) with semi-skimmed milk (Sainsbury’s, UK). The total energy content of the meal represented approximately 65 % RMR and comprised 39 % calories from carbohydrate, 54 % calories from fats and 7 % calories from protein. The exact composition of the meal was designed to ensure that an average 80 kg man with an RMR of 1791 kcal/day (estimated from Schofield equation; [16] received 1.5 g/kg carbohydrate [17]. A worked example showing exact proportions of each meal item is available in the supplementary information online. During the trial, participants were free to consume water ad libitum but consumed no further food until the end of the trial.

The standardized mixed meal with a high carbohydrate and fat content was intended to produce a more ‘physiological’ response compared with glucose or fat only challenges [18]. The meal was given relative to each individual’s resting metabolic rate to standardize energy intake for inter-individual differences in body mass/composition and thus resting energy requirements.

#### Blood and adipose sampling before and after meal consumption

Venous blood samples were taken from an antecubital forearm vein via a cannula and dispensed into separate tubes containing either K_3_EDTA or serum separation beads (Sarstedt Ltd., Leicester, UK) for plasma and serum separation, respectively. Samples for plasma separation were immediately centrifuged (3465 g for 10 min at 4 °C), whereas serum tubes were left to clot for 45 min prior to centrifugation. Subcutaneous adipose tissue samples (~1 g) were obtained under local anaesthetic (1 % lidocaine) approximately 5 cm lateral to the umbilicus using a ‘needle aspiration’ technique [19].

After baseline blood and adipose tissue sampling, the meal was consumed within 15 min. Further blood samples were taken from the cannula at 15, 30, 60 and 90 min and then at every hour until 6 h after consumption of the meal. A second adipose sample was also taken at 6 h on the contralateral side of the umbilicus to the sample collected at baseline to allow time for measureable changes to occur following peaks in both blood glucose and triglycerides following consumption of the meal. At both sampling time points, approximately 200 mg adipose tissue was homogenized in Trizol (Invitrogen, Paisley, UK) and processed for gene expression analysis and small portions cultured for analysis of adipokine secretion *ex vivo* over 3 h [7]. For tissue culture, adipose tissue was minced into ~5–10 mg explants and incubated with endothelial cell basal media (Promocell, Germany) supplemented with 0.1 % fatty acid-free bovine serum albumin, 100 U/mL penicillin and 0.1 mg/mL streptomycin (Sigma-Aldrich, Gillingham, UK). Tissue was incubated at a final concentration of approximately 100 mg tissue per 1 mL in duplicate for 3 h at 37 °C, 5 % CO_2_ and 95 ± 5 % relative humidity (MCO-18A1C CO_2_ incubator; Sanyo, Japan). Adipokine secretion from adipose tissue explants was corrected for the mass of tissue cultured and adjusted to central fat mass (L1-L4 from DEXA) as described previously [7].

### RT-PCR

Total RNA was extracted from whole adipose tissue using the RNeasy mini kit (Qiagen, Crawley, UK). Samples were quantified (Qubit 2.0 fluorimeter, Life Technologies, Paisley, UK) and 2 μg reverse transcribed to cDNA using a high-capacity reverse transcription kit (Applied Biosystems, Warrington, UK). Real-time PCR was performed using a StepOne™ (Applied Biosystems) using pre-designed primers and probes obtained from Applied Biosystems for measurement of glucose transporter type 4 (GLUT4; Hs00168966_m1), insulin receptor substrate 2 (IRS2; Hs00275843_s1), hormone-sensitive lipase (HSL; Hs00193510_m1), leptin (Hs00174877_m1), adiponectin (Hs00605917_m1), monocyte chemoattractant protein 1 (MCP-1; Hs00234140_m1), interferon gamma-induced protein 10 (IP-10; Hs01124251_g1), interleukin 6 (IL-6; Hs00985639_m1), interleukin 8 (IL-8; Hs99999034_m1), interleukin 10 (IL-10; Hs00961619_m1), interleukin 1 receptor antagonist (IL-1Ra; Hs00893626_m1), tumour necrosis factor alpha (TNF-α; Hs99999043_m1), interleukin 1 beta (IL-1β; Hs01555410_m1) and interleukin 18 (IL-18; Hs00155517_m1) expression. Adipose tissue expression of GCSF, MIP-1b and IFN-ɣ was also measured, but data are not shown because they were only detectable in 4–8 individuals. Peptidylprolyl isomerase A (PPIA/cyclophilin A) was used as an endogenous control [20]. Results were analysed using the comparative Ct method and expression normalized to an internal calibrator specific to each gene using the formula $2^{{ - {\Delta \Delta }C_{\text{T}} }}$ (where ∆∆*C*_T_ is [(*C*_T_ gene of interest − *C*_T_ PPIA) − lowest ∆*C*_T_ for gene of interest], and statistical analysis was performed on LN-transformed values [21].

### Biochemical analysis

Serum insulin was measured by ELISA (Mercodia, Uppsala, Sweden), and plasma glucose, serum total cholesterol, HDL cholesterol, ALT and triglycerides were measured using commercially available assay kits and analyser (Daytona Rx, Randox, Crumlin, UK). Adipose tissue secretion of IL-6, IL-8, IL-10, IL-1Ra, IP-10, GCSF, MCP-1, MIP-1b and TNF-α was measured using Luminex (Bio-Rad, Hercules, CA, USA).

### Statistical analysis

All data are presented as mean ± standard error of the mean (SEM). Total area under the curve (AUC) for insulin, glucose, triglycerides and NEFA was calculated using the trapezium rule and homoeostasis model assessment for insulin resistance (HOMA-IR) calculated using the formula fasting glucose (mmol/L) × fasting insulin (mU/L)/22.5 [22]. Adipose tissue responses to the meal were compared between the three groups using mixed-model ANOVA (adiposity × time) irrespective of minor deviations from a normal distribution within the normal physiological range [23]. Greenhouse–Geisser corrections were applied to intra-individual contrasts where *ε* < 0.75; however, for less severe asphericity the Huynh–Feldt correction was selected [24]. Where mixed-model ANOVA identified significant interactions effects, multiple *t* tests were applied to identify the location of variance both within each group relative to baseline and between groups at level time points, with *p* values subject to a Holm–Bonferroni correction [24]. Statistical analysis was performed using SPSS version 20 (IBM, Armonk, NY, USA) using an alpha level of *p* ≤ 0.05. The symbols used to identify significant differences are as follows: * denotes a main effect of time, † denotes a main effect of adiposity and # denotes any adiposity × time interaction effects.

## Results

### Fasting and postprandial blood measures of glucose and lipid metabolism

**Fig. 1:**
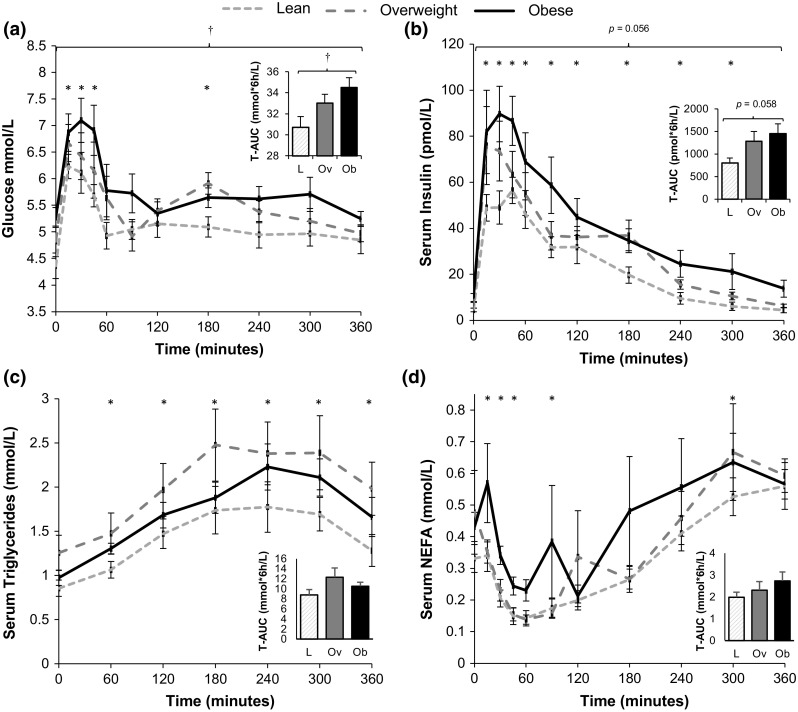
Blood glucose, insulin, triglyceride and NEFA responses over the 6 h following consumption of the meal for lean, overweight and obese individuals classified based on waist circumference. Temporal responses and total area under the curves (T-AUC) for **a** plasma glucose, **b** serum insulin, **c** serum triglycerides and **d** serum NEFA (*n* = 30). Mixed-model ANOVAs were performed with post hoc *t* tests applied to identify which specific time points were different from baseline; * denotes effect of time (*p* < 0.05) compared with *t* = 0; † denotes main effect of adiposity (*p* < 0.05). Total AUC compared using one-way ANOVA and *p* values shown. *L* lean, *Ov* overweight, *Ob* obese

Temporal insulin and glucose responses following the meal and their respective AUCs were elevated with increasing magnitude in proportion to increased adiposity (Fig. 1a, b). No such differences were apparent, however, for the temporal responses or AUCs for NEFA or triglycerides (Fig. 1c, d).

### Changes in adipose tissue gene expression at 6 h following the meal

**Fig. 2:**
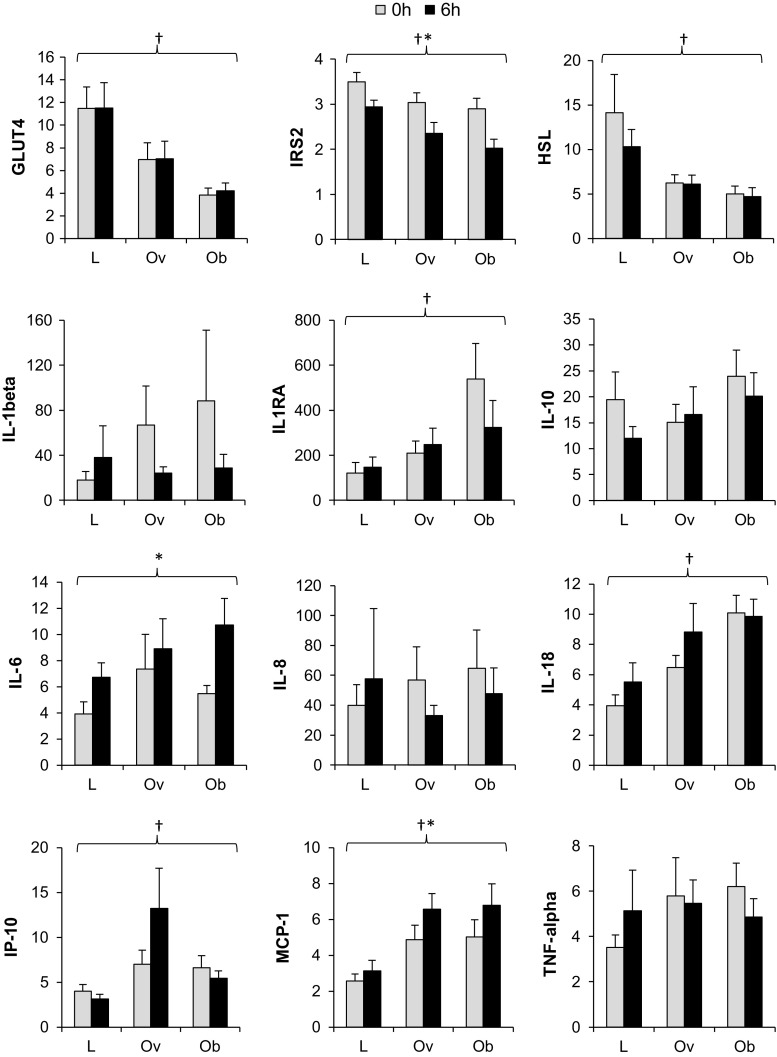
Relative gene expression in whole adipose tissue samples before and 6 h after consumption of a meal. Data presented as mean (SEM) with participants classified equally based on waist circumference. Expression is relative to a housekeeping gene (cyclophilin A/PPIA) and an internal calibrator specific to each gene and statistics performed on LN-transformed values. Groups were compared by mixed-model ANOVA; † denotes main effect of adiposity; * denotes significant main effect of time (*p* < 0.05) across the three groups. There were no significant adiposity × time interaction effects. *Note* IL-1β *n* = 27 after 2 outliers from IL-1β data excluded as >Q3 + (1.5 × IQR). Any samples above the threshold value (*C*_t_ > 35) were deemed undetectable and excluded from analysis. For all genes, *n* = 30 except where samples were outside the detectable limit *C*_t_ > 35 and were excluded from analysis; IL-10; *n* = 14 (lean *n* = 2, overweight *n* = 6, obese *n* = 6), IL-6; *n* = 28 (lean *n* = 8, overweight *n* = 10, obese *n* = 10), IL-8; *n* = 26 (lean *n* = 6, overweight *n* = 10, obese *n* = 10). *L* lean, *Ov* overweight, *Ob* obese

At 6 h after the meal, significant time effects reflected an up-regulation of IL-6 (*F* = 14.7, *p* = 0.001) and MCP-1 (*F* = 10.7, *p* = 0.003) and down-regulation of IRS2 (*F* = 24.6, *p* < 0.001) gene expression across all groups relative to baseline (Fig. 2). Greater expression of IL-18 (*F* = 8.3, *p* = 0.002), IP-10 (*F* = 4.1, *p* = 0.029), IL-1Ra (*F* = 6.1, *p* = 0.006) and MCP-1 (*F* = 8.8, *p* = 0.001) and lower expression of GLUT4 (*F* = 9.9, *p* = 0.001), IRS2 (*F* = 5.2, *p* = 0.012) and HSL (*F* = 4.8, *p* = 0.016) with increased adiposity were maintained over the 6-h postprandial period monitored in subcutaneous adipose tissue (Fig. 2). No differences in magnitude or direction of these responses were identified between groups (i.e. no adiposity × time interactions were apparent).

### Adipose tissue secretion before and after the meal

Baseline secretion of adipokines from adipose tissue together with changes following the meal is given in Table 2. Despite changes in gene expression of IL-6 and MCP-1 at 6 h following the meal, no significant changes in secretion were found. Adiposity × time interactions were identified for IP-10 (*F* = 4.3, *p* = 0.029) and IL-10 (*F* = 5.2, p = 0.017); however, post hoc *t* tests only indicated that IL-10 secretion from the obese adipose tissue was reduced to a level similar to the overweight group at 6 h (*p* < 0.05). Effects of adiposity on secretion were maintained across both time points for IL-6, MCP-1, GCSF, IP-10, MIP-1b and IL-10. Due to the limited size of some adipose tissue samples obtained, there was only sufficient material to culture tissue for six lean and five overweight individuals.

**Table 2:** Adipokine secretion by whole adipose tissue explants over 3 h, pre- and 6 h post-meal

	Lean (*n* = 6)			Overweight (*n* = 5)			Obese (*n* = 10)		
	Baseline secretion ng/3 h/estimated central fat mass	Secretion at 6 h ng/3 h/estimated central fat mass	Δ secretion from baseline ng/3 h/estimated central fat mass	Baseline secretion ng/3 h/estimated central fat mass	Secretion at 6 h ng/3 h/estimated central fat mass	Δ secretion from baseline ng/3 h/estimated central fat mass	Baseline secretion ng/3 h/estimated central fat mass	Secretion at 6 h ng/3 h/estimated central fat mass	Δ secretion from baseline ng/3 h/estimated central fat mass
IL-6^†^	2672 (543)	6517 (1977)^I^	3845 (−1014, 8703)	16,776 (5295)	14,035 (3222)	−2741 (−14,491, 9010)	15,573 (2091)	15,982 (1591)	408 (−4837, 5654)
MCP1^†^	1985 (380)	2381 (774)	396 (−1305, 2097)	7033 (1588)	5260 (598)	−1773 (−6027, 2481)	8155 (1298)	6969 (1242)	−1186 (−4241, 1869)
G-CSF^†^	487 (112)	987 (439)	500 (−481, 1480)	1763 (320)	1481 (232)	−283 (−1409, 843)	2133 (285)	1876 (195)	−257 (−1028, 515)
IL-8	463 (208)	1144 (505)^I^	681 (−628, 1989)	2479 (695)	1481 (346)	−998 (−3686, 1691)	3414 (1450)	1614 (517)	−1799 (−3990, 391)
IP-10^†^	639 (183)	1278 (578)	639 (−392, 1671)	3746 (770)	2238 (650)^I^	−1508 (−3378, 363)	3867 (709)	3251 (631)	−616 (−1538, 306)
IL-1Ra	167 (73)	161 (60)	−5 (−257, 247)	279 (40)	763 (417)	483 (−691, 1658)	1418 (563)	626 (172)^I^	−791 (−1722, 139)
MIP-1b^†^	220 (29)	401 (167)	181 (−246, 608)	1119 (101)	852 (143)	−267 (−879, 345)	1180 (194)	1341 (279)	161 (−444, 766)
TNF-α	230 (110)	180 (87)	−50 (−224, 124)	130 (15)	221 (107)	91 (−220, 401)	450 (182)	355 (135)	−95 (−229, 39)
IL-10^†^	14 (2)	12 (3)	−2 (−12, 8)	24 (2)	35 (9)	11 (−12, 34)	55 (8)	43 (8)*	−12 (−20, −3)

Participants were classified as lean (*n* = 6), overweight (*n* = 5) or obese (*n* = 10) based on waist circumference. Mean (SEM) values shown at baseline, with delta change and 95 % confidence intervals (CI) for the response within each group. Effects of adiposity, time and adiposity × time interactions analysed by mixed-model ANOVA

^†^Overall main effect of adiposity across both time points

* Time effect *p* < 0.05

^I^Time effect *p* > 0.05 but *p* < 0.1

## Discussion

The present study investigated systemic and adipose tissue metabolic and immune responses to a mixed meal in men with varying levels of adiposity. Although the magnitude of glucose and insulin responses to the meal were proportionate to increased levels of adiposity, up-regulation of IL-6 and MCP-1 and down-regulation of IRS2 gene expression in subcutaneous adipose tissue occurred to a similar extent in lean through to obese men 6 h following ingestion of the meal.

### Postprandial responses in adipose tissue with adiposity and insulin resistance

Within adipose tissue, gene expression of the typically proinflammatory cytokines IL-6 and MCP-1 were increased 6 h following consumption of the meal, which supports previous findings from people with metabolic syndrome [4] and non-obese relatives of people with type 2 diabetes [5]. The magnitude of changes in IL-6, MCP-1 and IRS2 gene expression observed in the present study was equal across all groups from lean to class I obese despite poorer glucose control/poorer insulin sensitivity with increasing levels of adiposity and despite participants with increased adiposity receiving a larger meal (since the meal was given relative to RMR). This indicates that these responses may be part of a ‘normal’ postprandial response within adipose tissue, at least within 6 h following a meal. A possible explanation for the similar responses is that relative insulin resistance in obese adipose tissue (suggested by the chronic down-regulation of GLUT4, HSL and IRS2) may counteract the increased insulin response to the meal and result in the same overall effect as in lean ‘insulin-sensitive’ tissue. Furthermore, given the increased relative mass of adipose tissue in obese individuals compared with lean, exposure of each gram of adipose tissue to insulin and glucose may actually be comparable.

### Potential stimuli of gene expression changes in adipose tissue

The meal used in this study was relatively high in both carbohydrates and fats. However, it cannot be determined from this study whether either of these or another factor related to the ingestion of either of these meal components (e.g. insulin) is the main stimulus for the increased gene expression of inflammatory cytokines in adipose tissue. Certainly, there is good evidence that insulin infusion alone increases IL-6 and TNF-α expression in subcutaneous adipose tissue from lean men [25, 26] and expression of IL-6, MCP-1 and IL-1β in rodents [27]. The effect of 3 h of hyperglycaemia alone (in the absence of increased insulin) on adipose tissue gene expression has also been examined [28], and although IL-6 and MCP-1 were not reported to be affected [28], this does not exclude glucose as a stimulus for the gene expression changes observed since different sampling time points were used. Assuming that the meal was indeed the key stimulus for changes in adipose tissue gene expression, another component to consider is triglycerides which, like the changes in adipose tissue gene expression, showed no differences in magnitudes of response with increased levels of adiposity. High-fat meal challenges have been shown to induce inflammation in adipose tissue [4, 29], and the type of fat (i.e. saturated or unsaturated) is not thought to be important in determining the extent of postprandial inflammation in adipose tissue [4]. It is possible, however, that adipose tissue may respond to glucose, insulin and triglycerides (and other associated stimuli not examined in this study, e.g. LPS) independent of each other, and adipose tissue sampling at different time points may have produced a different pattern of results. Although the exact stimuli would be difficult to isolate, understanding the dynamics of postprandial inflammatory responses in adipose tissue and how they relate to glucose control may ultimately provide insight regarding the aetiology of obesity-related insulin resistance.

### Potential origin and roles of postprandial cytokine responses in adipose tissue

Cells within the adipose tissue stromavascular fraction, in particular macrophages, are likely to be the major sources of adipokines such as IL-6 and MCP-1 [30–32], although mature adipocytes may also make a substantive contribution [33]. Each component of the meal and the associated insulin response have been shown in vitro to affect inflammation in both macrophages and adipocytes. For example, in experiments using (murine) 3T3-L1 adipocytes, insulin can dramatically enhance MCP-1 expression from these cells [34]. MCP-1 may be an important factor enhancing macrophage recruitment to adipose tissue (via repeated stimulation), as is observed in adipose tissue of obese individuals [35–38], and can also induce insulin resistance in adipocytes [34]. In vitro work has shown that IL-6 production can be induced in macrophages by high levels of glucose uptake via GLUT1 [39] and by fatty acids and lipopolysaccharides produced by gram-negative bacteria present within the intestine following ingestion of triglycerides [40–42]. Although chronic elevation of IL-6 is typically regarded as detrimental to insulin resistance and cardiovascular health [43], acute oscillations in IL-6, as seen for example following exercise, may be important in maintaining insulin sensitivity in muscle post-exercise [44, 45]. The implications of a postprandial increase in IL-6 gene expression within adipose tissue and its time course may therefore warrant further investigation.

### Considerations and future work

Cytokine secretion from adipose tissue remained similar in samples taken before and 6 h after the meal, which could be interpreted as evidence that adipose tissue has ‘protective’ mechanisms to prevent acute increases in the secretion of these cytokines in response to changes in gene expression (e.g. into the circulation). There appears to be large variation in postprandial secretion between participants, and this may reflect the wide ranges in adipose tissue responsiveness to the meal components and/or insulin and differences in relative proportions of adipocytes/non-adipocyte cells in adipose tissue. These results are limited to some extent by the small amount of tissue samples and data available for lean and overweight participants. Arterio-venous sampling may instead give a better indication of adipose tissue secretion over the entire duration following the meal and any effects of adiposity.

Based on this study design, we cannot be absolutely certain that the meal directly caused the reported time-related changes. Ideally, we would have been able to include some kind of comparator trial in order to rule out other explanations such as circadian rhythms. Such comparisons have been made previously in rats, which demonstrated that gene expression of inflammatory markers IL-6 and NF-KB was significantly increased (at 2 h) specifically following a high-fat meal compared with water [29]. However, even with such a study design it is difficult to conclude that the changes are due to feeding because of course extended fasting *per se* also represents a physiological challenge. Furthermore, it must be recognized that interpretation based on just two time points may not necessarily provide a full and valid reflection of the time course of events in vivo over the entire postprandial period. For example, there may have been insufficient time for synthesis of RNA transcripts/proteins for secretion into the circulation by the 6-h time point, or some proteins may already have been released from storage vesicles. In this context, a number of changes in gene expression have been identified at 4 h following lipid challenges [4] and after only 3 h of hyperglycaemia [28] and 2 h of hyperinsulinaemia [26, 27]. Repeated adipose biopsies to generate a time course would be useful but very challenging. It would also be interesting to investigate changes in adipose tissue following subsequent food intake, since blood glucose and insulin responses are known to be much lower after repeated feeding (i.e. Staub–Traugott effect) [46–48]. This could also help identify whether glucose and insulin are indeed key drivers of the postprandial adipose tissue responses.

## Conclusions

Although postprandial blood glucose and insulin responses are elevated with increasing adiposity, subcutaneous adipose tissue from lean through to class I obese individuals shows similar postprandial inflammatory responses at the gene expression level. Postprandial inflammatory responses in adipose tissue therefore do not appear to be related to the greater postprandial metabolic response (i.e. hyperinsulinaemia) observed with increased adiposity. This may be part of a normal adipose tissue response to feeding, or a ‘normalized’ response due to either reduced responsiveness of adipose tissue to meal components (e.g. glucose/lipid) or insulin or similar net exposure per gram adipose tissue (i.e. after adjustment for differences in adipose tissue mass).

## Electronic supplementary material

Below is the link to the electronic supplementary material.

Supplementary material 1 (PDF 60 kb)
