# Supplementary material for: Adipose tissue metabolic and inflammatory responses to a mixed meal in lean, overweight and obese men
Source: Eur J Nutr. 2015 Oct 29;56(1):375–85. doi: 10.1007/s00394-015-1087-7 (PMC5290042; doi:10.1007/s00394-015-1087-7)
Supplement: Supplementary file 1 — Supplementary material 1 (PDF 60 kb) [file 394_2015_1087_MOESM1_ESM.pdf]

Supplementary information

Article title: Adipose tissue metabolic and inflammatory responses to a mixed meal in lean, overweight and obese men

Journal name: European Journal of Nutrition

Author names and affiliations: RL Travers[1], AC Motta[2], JA Betts[1] and D Thompson[1]

[1]Department for Health, University of Bath, Bath, BA2 7AY, UK

[2]Unilever Food & Health Research Institute, Vlaardingen, The Netherlands

Address correspondence to: d.thompson@bath.ac.uk

Supplementary Figure: Examples of Excel Spreadsheet used to calculate meal composition relative to resting metabolic rate. A standard meal comprising brioche, jam, margarine and milkshake (icing sugar, cream, chocolate milk) plus milk (for tea) was devised for an average man of 80 kg to comprise the required total 120g CHO (1.5g/kg body mass). The relative ratios of each meal component (i.e. how much each item contributes to total CHO content) in this optimal meal were then used to ensure that these meal components would be in the same ratios when meal is relative to RMR.

Box A

Nutritional information per 100g →

		CHO	kCal CHO	fat	kCal fat	protein	kCal protein	Total calories
**brioche**	100g	55.5	208	11	98	9	36	342
**Stork -wrapped**	100g	0	0	75	668	0	0	668
**jam**	100g	62.4	234	0.1	1	0.5	2	237
**milk**	100g	5	19	1.7	15	3.4	14	48
**Sugar**	100g	100	375	0	0	0	0	375
**Choc milk**	100g	11.1	42	1.9	17	3.4	14	72
**cream**	100g	3	11	38.9	346	1.9	8	365

Box B

Nutritional information per optimal serving size →

	Serving size	CHO	kCal CHO	fat	kCal fat	protein	kCal protein	Total calories
**brioche**	88	49	183	10	86	8	32	301
**jam**	40	25	94	0	0	0	1	95
**stork**	38	0	0	29	254	0	0	254
**Icing sugar**	16	16	60	0	0	0	0	60
**milk**	50	3	9	1	8	2	7	24
**cream**	70	2	8	27	242	1	5	256
**choc milk**	235	26	98	4	40	8	32	170
**total:**	**537**	**120**	**452**	**71**	**630**	**19**	**77**	**1158**
%			**39**		**54**		**7**	

Box C

CHO required calculated from CHO ratio (Box E) →

Total to give = CHO to give (Box C)/CHO per 100g (Box A)*100

All other nutritional info calculated from total to give and nutritional info per 100g (Box A)

	Totals to give	CHO	kcal CHO	fat	kcal fat	protein	kcal protein	total calories
**Brioche**	**84**	47	175	9	82	8	30	288
**jam**	**38**	24	90	0	0	0	1	91
**stork**	**36**	0	0	27	243	0	0	243
**Icing sugar**	**15**	15	57	0	0	0	0	57
**milk**	**48**	2	9	1	7	2	7	23
**tea**	**192**							
**cream**	**67**	2	8	26	232	1	5	245
**choc milk**	**225**	25	94	4	38	8	31	162
	**Totals:**	**115**	**433**	**68**	**603**	**18**	**73**	**1109**
			**39**		**54**		**7**	

Box D

**Person data**	
RMR	1715
CHO/kcal RMR	0.067001675
CHO reqd	115

← RMR entered and CHO required calculated automatically based on required ratio

Box E

	CHO Ratios	
**Brioche**	2.46	
**jam**	4.8	
**Icing sugar**	7.5	
**milk**	48.0	
**Choc milk**	4.6	
**Stork**	0.432	Given relative to Brioche
**cream**	0.298	Given relative to choc milk

(Brioche to Choc milk) = CHO required (Box D) /CHO in optimal serving size for each item (Box B)

Assumptions:

Average man of 80kg has a resting metabolic rate (RMR) of 1791 kcal/day (Schofield equation).

For a high carbohydrate input, the meal should contain1.5g CHO/kg body mass (Betts…).

An 80 kg man therefore receives 120 g carbohydrate. CHO per kcal RMR is therefore 120/1791 = 0.0670391……

Therefore, CHO required per person = RMR x 0.0670391…….
